# Address to the Graduates of the Ohio College of Dental Surgery

**Published:** 1874-05

**Authors:** G. W. Keely

**Affiliations:** Oxford, O.


### ADDRESS TO THE GRADUATES OF THE OHIO COLLEGE OF DENTAL SURGERY FEBRUARY 24th, 1874.

BY G. W. KEELY, D. D. S., OXFORD, O.

*Young Gentlemen:*—With the exercises of this evening, your course of study in the Ohio College of Dental Surgery terminates. This is your commencement. Webster says, it is the day when degrees are confered by colleges and universities upon students and others.

Since the begining of your pupilage, you have no doubt looked forward to this day with bright anticipations. And, now, it will not be long before you will enter upon the active duties, responsibilities, and stirring scenes of life.

If any one of you is vain enough, to suppose that your education is complete, upon the reception of a diploma, and bidding farewell to these halls, the sooner the scales are removed from your eyes and the delusions dispelled, the better. You have but laid the first stones in the foundation, upon which the edifice of professional success may be built.

It is but the seed sown; and if good seed, in rich ground, and industriously cultivated, will surely produce an abundant harvest. In pursuing your studies here, your minds have been trained in the right direction, and as it is impossible for you to stand still, you must go forward, or take the retrograde course, if the latter, it would be better had you never entered this institution.

It is certainly true, that a diploma, without any further study whatever, will enable you to be quite an accomplished humbug, an ingenious surface skimmer superficial in almost everything, ripe, substantial, and thorough in nothing.

Every one has heard of learned fools, in medicine, law, ministry, as well as in our own profession. They are of that class who get a diploma, and then come to the conclusion they know it all, their education being complete, they shut down the brakes, and stop.

Far better for all such, and those with whom they come in contact, had they never made a beginning.

This class you may meet in the medical and dental profession, and they always refuse to get out of their shell.

In diagnosing a case, they are generally as wise as an intelligent owl, and by skillful maneuvering, manage to deceive their innocent patients.

This is especially true of the medical man, who points with pride to his diploma, in a magnificent gilt frame, hanging in a conspicuous place on the wall, in his office; and if he is the greatest, and most consummate dolt in christendom, can get some to partake of his drugs, and die under the affliction of medical ignorance. Such a man will as a matter of course, ignore the journals of the day; why need he be posted in regard to new remedies and the most approved methods of treating disease, when he has a soverign remedy peculiar to himself (he being a Doctor by nature) for every disease, to which humanity is heir. Every case will be of a serious nature and he will tell you of his numerous patients cured in less than no time, after many of the most eminent physicians had labored hard and exhausted their skill to no effect.

As for medical societies, they are humbugs of the first water; and he does not propose to waste his precious time in attending them, and imparting information that would make others, as wise as himself, without a dollar and cent consideraticn. The fee bill is the insurmountable objection. As a conscientious and Christian gentleman, he can't pledge himself to charge exorbitant fees for his services, as is necessary for those who spend so much time in attending such meetings.

It is easy to say this man will soon find his level, and rust out for want of practice, but such is not always the case, for we find in every community, many of the most intelligent, and wealthy, encouraging this class of empirics, who grow rich on this ill gotten gain.

He may commit many of the most egregious blunders, and if questioned concerning them, will tell you, that he is not the first great man, who made a mistake. In fact after the most scientific examination, he may diagnose an ovarian tumor; and as each month passes by, express his surprise at its rapid growth, and if a suggestion is offered by patient, or friends, of the bare possibility of a mistake, he will wisely shake his head, and settle the question for that time, by asserting the impossibility of his being in error. But as time and tide waits for no one man, in all human probability this case will run its natural course, resulting not in a fibrous or fluid tumor, but bringing to light, to the great joy of the anxious parents, a human responsibility, and this will be the result so long as the bold and impudent charlatan, seizes upon the weakest elements of the human mind, and rests his success on the blind faith, and credulity of his dupes.

As much as this is to be deplored, it will exist in the absence of a recognition of medical science by government, and a proper appreciation by the people, of the truly educated physician.

The medical profession of to day understands full well, that their only hope is to look exclusively to the most ample, and complete qualifications.

Young gentlemen: the profession you are about to enter is a speciality of medicine, and you must make up your minds to meet the responsibilities resting upon you.

For your encouragement, I would most gladly say to you, that there are quacks in all professions but ours. The contrary being the case, we must look the matter squarely in the face. To a close observer it really seems that the great mass of American people, are anxious to be humbugged. In our great cities, as in the more rural districts, a man after a few weeks pupilage, may tack up his shingle, and on it in letters of gold, "John Smith Surgeon and Mechanical Dentist," and high above it he hangs a huge banner on the outer wall, and on it in black letters a yard long the pedestrian is informed. Here is the place to get a set of teeth for ten dollars worth thirty, teeth filled with gold for one dollar worth five to ten, and per chance as an additional inducement, a mammoth set of wooden teeth covered all over with gold leaf, swings over his office door.

The daily Journals are also used as a vehicle to herald his fame to the uttermost parts of the earth.

For a time, at least, patients come by scores not only from the poor and middle classes but the wife of the millionaire in fine carriage with driver and footman, puts in an appearance and secures the services of this eminent practitioner.

And she thinks it wonderful with what ease, and rapidity, her teeth are put in perfect order and how foolish she had been, for anticipating and dreading the pain of the operation for none had been inflicted.

The moderate charge is willingly paid with many grateful thanks, and she goes to her mansion, and anxiously awaits the return of her husband, to whom she relates her good fortune, and what a jewel of a man she had found in Dr. John Smith, surgeon dentist.

Such characters can not conceive of any earthly use for a private pupilage of two or three years, and a regular course in a dental college, where they learned to make a set of teeth and fill teeth as good as any other man in a few weeks.

They have no magnetic attraction in the direction of dental societies, and consequently they are wholly ignored, and considered unworthy of attention.

We would naturally look for better things from a graduate (even if his diploma was obtained under false pretenses,) but some of these are found plodding along in the rut in which they first slipped, and seem totally ignorant and oblivious to the wonderful advancement made in dental science, suggest to them the use of the rubber dam, they will ask; what's that for, or speak of the dental engine what do you do with that? and so with almost every improvement, made within the last ten or fifteen years.

Not a year since, I enquired of one, if he had a Morrison eugine? Well no; I declare, I must look up my last *Cosmos*, and see a description of it. This remark discouraged me too much to speak of the rubber dam, and I consequently defered its mention untill some more auspicious occasion.

It is perhaps superfluous for me to say, that such a distinguished gentleman, is not a member of a dental association.

As for the use of the mallet, that's an old fogy institution by which many teeth have been pounded to death, split in twain, and otherwise permanently destroyed—and besides all this, it is a waste of the precious metal, packing so much gold from sight in a cavity.

And then it is a great waste of time, for he could insert six or eight fillings in his way while one filling was made by the use of the mallet. He knows this to be a fact, for in his fifteen year's practice, the mallet has never been used in his office and he has never witnessed its use in a single filling.

He instructs his patients to beware of the mallet, and all other new fangled contrivances, for inflicting unnecessary pain.

Not long since a lady called upon me, to have some fillings examined, I found nine very large amalgam fillings in her molars. She enquired, will they save my teeth? I told her, I feared not, as the fillings were very rough, unfinished and discolored, and looked as if done in great haste. She asked, "how long would it take you to fill them with the same material?" With the aid of my Morrison engine, I can prepare a cavity, in half the time it would require in the old way.

But I feel quite sure, I could not fill any one of them, in less than an hour. "But Doctor; you are not in earnest!" I explained the necessity of removing all the decay, and preparing the cavity, introducing the filling, and finishing it. 'But why does it take you so long, when mine were all filled in an hour and a half—and by a good dentist, for I saw his diploma hanging in his office, and he certainly does a great deal of work, and then, he is so kind and gentle. he didn't hurt me a bit.

And you know doctor, all your patients can't say that of you, and then you make appointments, and I am told, some have to wait for weeks. My dentist, does the work right away, and he makes lots of money too. Now, these teeth have been filled nearly three months, and not one has fallen out."

Gentlemen: I could not say much, how could I, under such embarrassing circumstances? however, I did venture the assertiun, that at times, it was necessary to inflict some pain, in making a good and permanent operation, and I had yet to learn the art of trotting my patients through at two forty speed, (she was the wife of a wealthy farmer, and a graduate of a female college.)

This state of things, exists all over the country, and is greatly to be deplored.

Here in the Queen City of the West, men calling themselves dentists, living almost within a stone's throw of this institution, have been plying their trade, and attending to numerous customers for years, and have never been known to come within these walls, or show their face, at a dental association.

But with the rapid advances now making in dental science, the increase in the number of schools, and dental societies, and the interest manifested by the best men in the profession, will have a tendency to bring about a better state of affairs.

The people are being rapidly educated up to the point of demanding the best operations, and the dentist who fails to fulfill these requirements, can not long, command the patronage of those, who are willing to pay for the best skill.

They will inquire, in regard to the young practitioners pupilage; at what college he graduated; as to his skill as an operator, his industrious habits, his moral worth, his application to business, and interest manifested in his chosen calling, by subscribing for the leading journals, and his attention and interest taken in associated action, at dental societies.

Here, you have been listening to lectures, and witnessing the various demonstrations pertaining to your future calling.

The lectures have embodied a great amount of teaching, and if you have been attentive, and in future industriously persue the studies here commenced, there is no reason, why you should not become distinguished practitioners.

If you have the gift of genius it is incumbent upon you to cultivate, and improve it to the highest degree possible, and if you fail to do this, you will prove recreant to the duty you owe God, humanity, and yourself. At once, set your mark high with a settled and rational determination to succeed, and much of the difficulty will at once be surmounted. Never say "I can't!" for its a naughty word—but go to work honestly energetically, and faithfully, and success will crown your efforts.

Industriously improve your time, and the talents God has given you, with a fixed purpose in the right course, and the community will feel, and acknowledge it, wherever your lot may be cast.

During the first few years of your professional life, you will have more time to study, and be in better condition to do so, than after a large practice is gained, for I assure you, that the life of the faithful dentist, is not one of ease, by any means. It is a hard, laborious life, a constant drain, both upon his mental and physical energies. If you have been induced to enter the profession with the hope of making money and rapidly accumulating a fortune, I would advise you to abandon it at once, for this is not the experience of any faithful dentist, in the land.

Do not, under any circumstances, be induced to slight an operation, in order to be the immediate possessor of a few paltry dollars, for in the long run, it will prove disastrous in more ways than one. Let your motto be "The money taken from a patient not honestly earned, will do me no good."

It takes time, patience, energy and perseverence to make first class operations, and he who sticks close to this text, will soon find but few idle hours, during the business portion of the day.

I would advise you to cultivate friendly relations with medical men, and quietly convince them, that you are worthy of recognition, and capable of consulting with them, particularly in regard to your department, of the healing art.

Do not be obsequious to them, nor operate for them at low figures, with the hope of their recommending you, for in this you will be disappointed.

I have tried the latter experiment, for the past quarter of a century, and have yet to realize the first dollar on my investment. Remember always "The laborer is worthy of his hire."

This is an age of rapid improvement in every scientific department, and intelligent patients have the right to expect and demand that you should be a man of science and general culture.

The confidence reposed in the dental surgeon, is so great that his success must depend greatly upon his being a man of moral worth, and the possesor of the strictest integrity, coupled with polite and gentlemanly deportment.

Your office should be well furnished and kept scrupulously neat and elean, and no undue familiarity ever indulged in with your patients.

Your instruments should be kept out of sight as much as possible for the time has gone by when display of this kind will be to your credit or advantage.

All of your instruments, chair and all other appliances should be of the best in the market, for the comfort of your patient as well as your own.

In the management of your practice you will soon find that a fair share of pluck is indispensable and if prudently employed will ere long prove an element of success.

Be honest, and dare to do right under all circumstances, never allowing the whims of a patient to direct you from the path of duty.

Make your operations in the best possible manner, and in the end you will reap an abundant harvest.

Be corteous, kind and lenient towards your competitors, that they may be compelled to acknowledge, that you are at least a gentleman.

Finally gentlemen, we welcome you to the ranks of our profession, your mission on earth is to do good.

Be faithful to your God, your patients, society and yourself, cultivate the virtues that survive the ravages of death between which and happiness there is no intervening cloud.

